# Sustainable urban systems: from landscape to ecological processes

**DOI:** 10.1186/s13717-022-00371-3

**Published:** 2022-03-10

**Authors:** Yuyu Zhou, Gang Chen, Weiqi Zhou

**Affiliations:** 1 Department of Geological and Atmospheric Sciences, Iowa State University, Ames, IA 50011 USA; 2 Laboratory for Remote Sensing and Environmental Change (LRSEC), Department of Geography and Earth Sciences, University of North Carolina at Charlotte, Charlotte, NC 28223 USA; 3 State Key Laboratory of Urban and Regional Ecology, Research Center for Eco-Environmental Sciences, Chinese Academy of Sciences, Beijing, 100085 China; 4 University of Chinese Academy of Sciences, Beijing, 100049 China

Our world is experiencing unprecedented land cover and land use changes (LCLUC) due to population growth, economic development, and global climate change. Urbanization, one major form of LCLUC, has received growing attention for its significant role in changing ecological processes and functions of urban landscapes. There is a growing need, from both the academic and the policy-making communities, for science-based information and evidence to enhance our understanding of the urban ecological processes and societal needs because of the rapid urbanization over the past few decades across the world. Such information is key to informing the development of best practices for improved landscape and urban planning. This special issue on *Ecological Processes* provides an update of methodologies and scientific progresses on urban ecological processes across multiple spatial and temporal scales. This issue includes nine papers in three focus areas: (1) mapping and modeling urban landscape; (2) monitoring urban environment and its dynamics, and (3) linking landscape and environmental changes to ecological processes.

## Mapping and modeling urban landscape

Urbanization has caused significant landscape changes in cities and their surrounding areas. Accurate mapping and forecasting of the heterogeneous land cover/use and its temporal dynamics is essential for evidence-based decision-making and effective urban management and environmental conservation. Over the past decades, remote sensing has gradually become a standard tool in urban LCLUC mapping. Particularly with the increasing availability of Earth Observation datasets, urban researchers and practitioners are now having a variety of options to complete the mapping task to address existing and emerging challenges. Earth Observation datasets feature large volumes and varying formats, and are collected by many platforms at different spatial/temporal scales. Mapping strategies and algorithms that have been used for the past five decades have to be adjusted or even redeveloped to meet the needs of handling massive datasets for accurate retrieval of urban LCLUC from local to global scales. For example, geographic object-based image analysis (GEOBIA or OBIA) has led to a well-recognized paradigm shift in high spatial resolution image classification since the early 2000s (Chen et al. 2018). In this special issue Qian et al. (2020) aims to address a similar challenge by developing a novel classification framework that integrates remotely sensed imagery, infrastructure data available at the municipal level, and GEOBIA to map the hierarchical spatial heterogeneity of urban landscapes.

While mapping current or historical LCLUC allows us to understand how urbanization has driven the change in ecological processes, forecasting LCLUC can inform effective planning to address ecological challenges in future urban development. Capitalizing on historical LCLUC and environmental factors, geospatial modeling aims to project where changes will possibly occur under various scenarios, e.g., using different planning strategies. A variety of LCLUC forecasting models have been developed over the past two decades, including the popular Cellular Automata (CA) models (Wang et al. 2021), with most models being statistical- or rule-based. To improve CA’s generalization ability, Li et al. (2020a) in this special issue proposed a Logistic-Trend-CA model, which was applied to simulate urban spatial sprawl in the Beijing–Tianjin–Hebei region of China. The proposed model significantly outperformed the traditional Logistic-CA model and was found to be more suitable for urban sprawl modeling over a long period.

## Monitoring urban environment and its dynamics

Urbanization also leads to significant changes in urban environment (e.g., thermal and atmospheric conditions) (Mohd Shafie et al. 2022; Chen et al. 2016). An improved understanding of urban environment within and across cities is highly needed for its implications in human activities and welfare. Although satellite observations play an important role in monitoring urban environment with huge heterogeneity, the missing values in these observations due to multiple factors (e.g., cloud) increase the challenges to understand urban environment and its dynamics. A variety of models and algorithms have been proposed to improve existing satellite observations or develop new indicators for monitoring urban environment. For example, with the advance of these methods, the gap-filled seamless data of land surface temperature and aerosol optical depth have been developed (Zhang et al. 2022; Li et al. 2020b), and derived products from satellite observations such as phenology indicators with high spatial resolutions in urban areas are emerging (Li et al. 2019). These improved products in terms of coverage or spatial resolution serve as important and complementary information to understand urban environment. In this special issue, Xia et al. (2020) investigated concentrations of a series of heavy metals in water and sediments from 20 lakes along a rural to urban gradient in central China and observed an increasing trend of metal concentration from rural to urban lakes. When studying the impact of the COVID-19 pandemic, Hallema et al. (2020) found a positive aspect of economic slowdown for improved water quality, and illustrated environmental resiliency and societal control over urban water quality.

Physically based modeling can not only fill the gaps in observations or derivatives from remote sensing and field measurements, but also provide capability to simulate the potential changes in urban environment under different scenarios. For example, the Weather Research and Forecasting (WRF) model, a non-hydrostatic numerical weather prediction system, has been widely used to investigate the impact of urban landscape on microclimate (Li et al. 2018). In addition to the WRF, a number of numerical models in microclimate modeling at different spatial scales have been developed to understand urban thermal environment. In this special issue, Teichmann et al. (2021) simplified the modeling process of the uhiSolver software—a microclimate simulation program, to help the investigation of the influence of different construction methods and ground surfaces on the Urban Heat Island effect.

## Linking landscape and environmental changes to ecological processes

The changes of urban landscape and environment associated with urbanization in cities and surrounding areas have been one of the major drivers for the changes in ecological processes, biodiversity, other functions, and the resident (Chen et al. 2016; Seto et al. 2012; Zhou et al. 2022). Numerous studies have shown that rapid urbanization directly drives the loss of wildland habitat and increases habitat fragmentation, and thereby threatens biodiversity (Seto et al. 2012). Urbanization also directly or indirectly changes the ecological processes of urban ecosystems, degrading urban ecosystems and resulting in adverse ecological and environmental impacts (Zhou et al. 2022). The impact of the changes in landscape structure (e.g., LCLU) on ecological processes and biodiversity have long been a research frontier in urban ecology, which will continue to be a research frontier in the context of rapid urbanization and changing climate.

In this special issue, several papers reported recent progress in linking landscape structure, ecological processes, and biodiversity in urban environments. Hakkoum et al. (2021) found that the input of organic matter and nutrients from the treated wastewater can increase the biodiversity of soil cyanobacteria, but municipal and mining solid wastes lead to decrease of diversity and microalgal biomass. Leveau (2021) found that environmental filtering by urbanization led to phylogenetical randomness and functional clustering of bird communities in urban habitats. By comparing nutrient cycling and litter decomposition between the litter of a single species and a mixed composition of litter in urban parks in São Paulo City, Brazil, Ferreira et al. (2021) found that both environmental and biogeographic characteristics of the species shall be considered in urban reforestation programs. Additionally, Liu et al. (2021) found that spatial processes and environmental filtering equally contributed to macroinvertebrate metacommunity dynamics in the highly urbanized subtropical river networks in Shenzhen, South China through examining ecological factors and the seasonal difference in structuring macroinvertebrates metacommunity.

The changes in urban landscape and the environment under unprecedented urbanization are expected to continue in the next few decades, and these changes will remain as major drivers to shape urban ecology in a complex way (Zhou et al. 2021). An improved understanding of these changes and their implications in urban ecology can significantly guide urban development and design toward sustainable urban systems. Finally, we would like to thank all authors and reviewers for their contribution to this special issue. We hope that this special section will inspire further advancement in the understanding of various aspects of urban ecological processes across multiple spatial and temporal scales for developing sustainable urban systems.
